# Trophic Relationships between Aphids and Their Primary Parasitoids

**DOI:** 10.1673/031.012.7801

**Published:** 2012-07-09

**Authors:** Imtinan A. Khan, Muhammad Naeem, Soaib A. Hassan, Hazrat Bilal, Ata-ul-Mohsin, Imran Bodlah

**Affiliations:** ^1^ Department of Medical Entomology and Disease Vector Control, Health Services Academy Islamabad Pakistan; ^2^ Department of Entomology, PMAS—Arid Agriculture University, Rawalpindi, Pakistan

**Keywords:** food web

## Abstract

The present research was carried out to study the trophic relationship between aphids and their primary parasitoids in Pothwar, Pakistan during 2009–2010 in the districts of Rawalpindi, Attock, Chakwal, and Jhelum. Ten species of aphids were recorded from 17 host plants. The aphids were parasitized by 11 species of primary parasitoids. Five quantitative aphid-parasitoid food webs were constructed describing the trophic relationships between the community of aphids and their primary parasitoids.

## Introduction

The relationship between plants and insects is one of coexistence; insects that are harmful to plants are suppressed by other insects (Scatter et al. 2001). Aphids are an extremely destructive group of insect pests that attack both agronomic and horticultural crops by sucking the sap of leaves and young shoots causing distortion, stunting, and sometimes premature leave fall (Dixon 1973; Akhtar and Khaliq 2003). Parasitoids represent an important element in the aphid environment, and about 400 species have been described worldwide (Stary 1988; Dolphine and Quick 2001). The majority of aphid parasitoids have been reported from four families of which one belongs to Diptera (Cecidomyiidae) and three to Hymenoptera (Braconidae, Aphelinidae, and Encyrtidae) (Raychaudhuri 1990). Insect parasitoids play a major role in terrestrial food webs as they are highly diverse, abundant, exploit a wide range of niches, and are capable of influencing the population densities and dynamics of their hosts (Hassell 2000). The food web explains the trophic relationships between the sets of interacting species and is thus the fundamental tool in community ecology (Polis and Winemiller 1996). The individual food web gives information about the potential direct and indirect dynamic interactions that link species within the community, while collections of the food webs offer the prospect of detecting patterns in the way natural communities are structured (Cohen 1989). The communities of hosts and parasitoids provide a good system for study using the quantitative food web because the trophic links between the hosts and parasitoids are relatively easy to establish and quantify (Lewis et al. 2002). The pattern of communities could be measured by constituent population, their range of distribution, and interactions between them. This pattern in the community composition is due to the abundance and the distribution of species inhabiting them (Yazdani and Agarwal 1997).

The aim of this study was to investigate the pattern of the aphid—parasitoid community in the field and the horticultural crops in the Pothwar region. A particular emphasis was placed on describing trophic relationship between the aphid and its primary parasitoids with the help of food webs. This work will contribute to a better understanding of the trophic relationships between different species of aphids and their primary parasitoids.

## Materials and Methods

**Figure 1.**
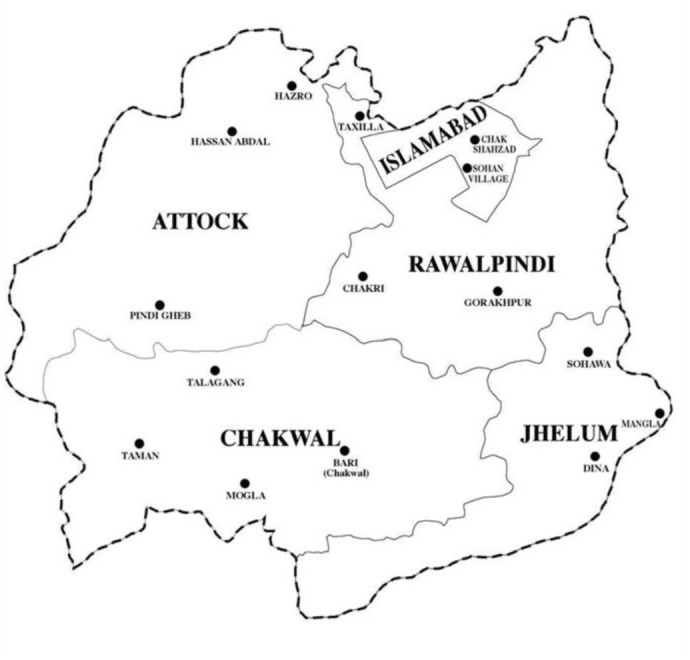
Map of Pothwar region showing the collection sites of aphids and primary parasitoids during 2009–2010. High quality figures are available online.

The survey was conducted during 2009–2010 from different districts within the Pothwar region (Rawalpindi, Attock, Jhelum, and Chakwal) (Figure 1). Aphids and aphid mummies were collected on each month from different field and horticultural crops including *Rumex acuta, Cypeus rotundus, Casia fistula, Solanum tuberosum, Brassica compestris, Brassica oleracea, Brassica oleracea var. botrytis, Raphanus satius, Brassica rapa, Zea mays, Triticum aestivum, Avena sativa, Hordeum vulgare, Hibiscus sp., Pisum sativum, Trigonella fenumgraecum,* and *Rosa sp.* The quantitative data was collected on the density of aphids and aphid's mummies per unit of their host plant.

Plants of each species were randomly selected and the number of aphids and aphid mummies were counted by using a 1 m^2^ quadrat. Aphid and parasitized aphid densities were made absolute by multiplying the number per plant unit to the estimated densities of plant per m^2^. The structure of the parasitoid community was assessed by rearing mummies collected in quantitative sampling programs. The aphids collected were identified up to the species level by using the key of Blackman and Eastop (1984).

The collected mummies were kept in glass vials. In the laboratory, mummified aphids were reared in the vials with a small piece of moistened cotton to keep appropriate moisture levels. The vials were carefully labeled and covered with a piece of muslin cloth to provide aeration in the vials. Parasitoids that emerged from mummies were killed by placing vials in the freezer for five minutes. The specimens were identified up to the species level using the key of Raychaudhuri (1990). The identification criteria were based on external morphology.

All the data were analyzed by using one—way ANOVA in SPSS® version 16.0. Microcal Origin® 6.0 was used for graphical representation of the results, and food webs were constructed by using Macromedia FreeHand 9.

## Results

**Figure 2.**
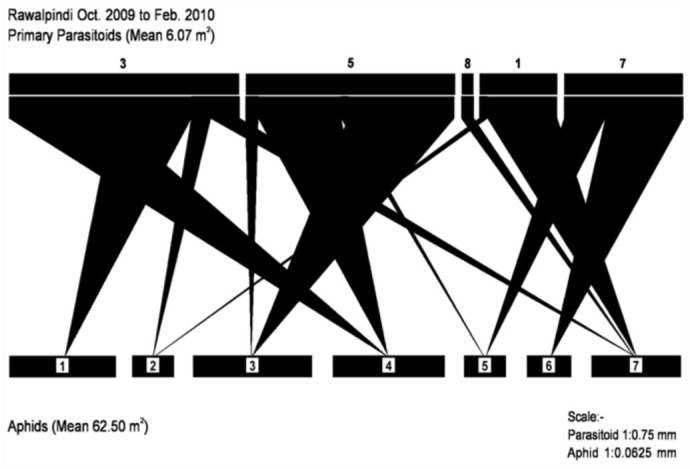
Aphid—parasitoid web in District Rawalpindi from October 2009 to February 2010. Primary parasitoids are shown above: (3) *Aphidius sp*, (5) *Diaretiella rapae*, (8) *Aphidius absenti*, (1) *Trioxys sp., (7) Aphidius colemani*. Aphid species are shown below: (1) *Aphis fabae*, (2) *Aphis gossypii*, (3) *Brevicoryne brassicae*, (4) *Myzus persicae,* (5) *Schizaphis graminum*, (6) *Rhopalosiphum padi*, (7) *Aphis craccivora*. High quality figures are available online.

**Figure 3.**
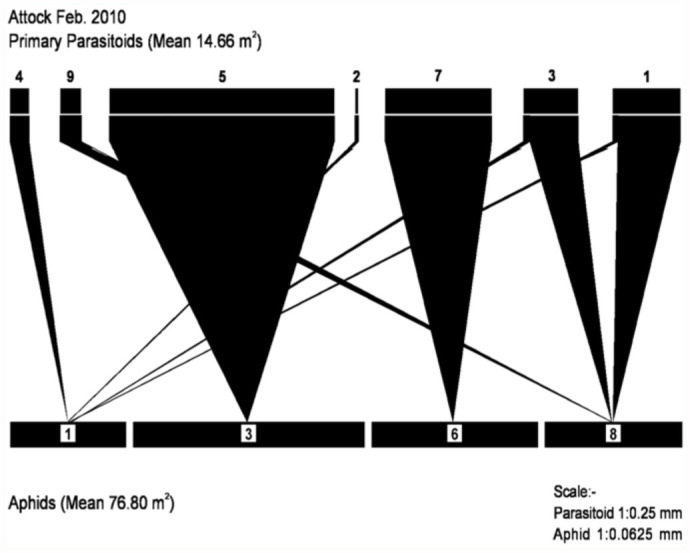
Aphid—parasitoid web in District Attock in February 2010. Primary parasitoids: (4) *Binodoxys indicus*, (9) *Aphidius rosae*, (5) *Diaretiella rapae*, (2) *Binodoxys anglicae*, (7) *Aphidius colemani*, (3) *Aphidius sp*., (1) *Trioxys sp.* Aphid species: (1) *Aphis fabae*, (3) *Brevicoryne brassicae*, (6) *Rhopalosiphum padi*, (8) *Lipaphis erysimi*. High quality figures are available online.

**Figure 4.**
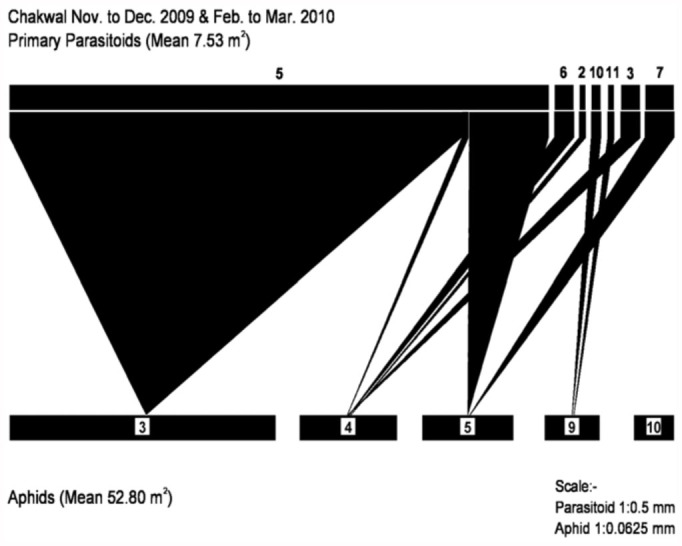
Aphid-parasitoid web in District Chakwal from November to December 2009 and February to March 2010. Primary parasitoids: (5) *Diaretiella rapae*, (6) *Aphidius ribis*, (2) *Binodoxys anglicae*, (10) *Aphidius smithi*, (11) *Aphidius urticae*, (3) *Aphidius sp*, (7) *Aphidius colemani*. Aphid species: (3) *Brevicoryne brassicae*, (4) *Myzus persicae*, (5) *Schizaphis graminum*, (9) *Acrythosiphon pisum*, (10) *Rhodobium porosum*. High quality figures are available online.

**Figure 5.**
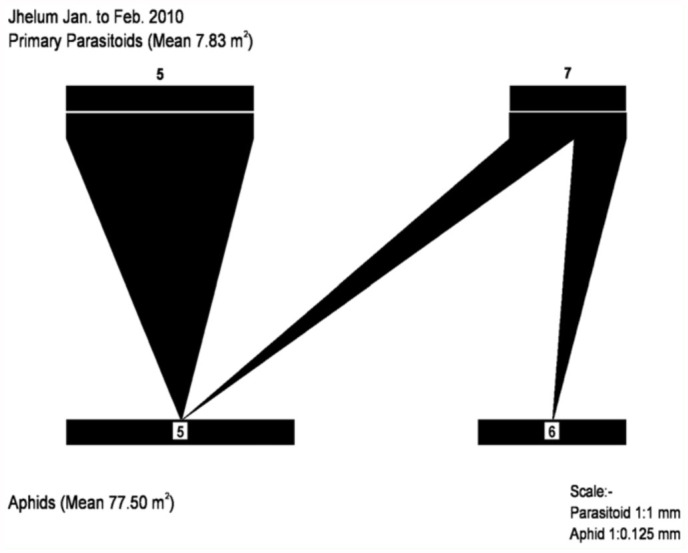
Aphid—parasitoid web in District Jhelum from January to February 2010. Primary parasitoids: (5) *Diaretiella rapae,* (7) *Aphidius colemani*. Aphid species: (5) *Schizaphis graminum*, (6) *Rhopalosiphum padi*. High quality figures are available online.

**Figure 6.**
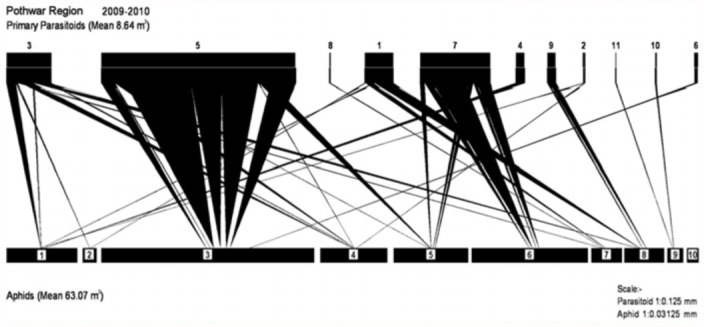
Aphid-parasitoid web in Pothwar region during 2009–2010. Primary parasitoids (3) *Aphidius sp*., (5) *Diaretiella rapae*, (8) *Aphidius absenti*, (1) *Trioxys sp*., (7) *Aphidius colemani*, (4) *Binodoxys indicus*, (9) *Aphidius rosae*, (2) *Binodoxys anglicae*, (11) *Aphidius urticae*, (10) *Aphidius smithi*, (6) *Aphidius ribis.* Aphid species: (1) *Aphis fabae*, (2) *Aphis gossypii*, (3) *Brevicoryne brassicae*, (4) *Myzus persicae*, (5) *Schizaphis graminum*, (6) *Rhopalosiphum padi*, (7) *Aphis craccivora*, (8) *Lipaphis erysimi*, (9) *Acrythosiphon pisum*, (10) *Rhodobium porosum*. High quality figures are available online.

Ten species of aphids were recorded from different localities of the Pothwar region (Figure 1). The species and host plants are given in Table 1. Aphids were attacked by 11 species of primary parasitoids. All the associations between aphids and their primary parasitoids that were established are listed in Table 2. Aphid colonies were found from October until March. Four aphid-parasitoid webs were constructed from data collected from Rawalpindi, Attock, Chakwal, and Jhelum districts based on the collection of aphids and their primary parasitoids during the months shown in Figures 2–5. The overall aphid—parasitoid food web in the Pothwar region from October 2009 to March 2010 is shown in Figure 6.

To understand how the aphid—parasitoid webs work, consider the example of the aphid—parasitoid web in district Rawalpindi from October 2009 to February 2010 (Figure 2). In these five months, seven aphid species were recorded from different localities of the district (*Aphis fabae, Aphis gossypii, Brevicoryne brassicae, Myzus persicae, Schizaphis graminum, Rhopalosiphum padi,* and *Aphis craccivora*), and their relative abundance is indicated by the widths of the seven numbered bars at the bottom of the diagram. As shown, the mean density of aphids collected from October 2009 to February 2010 was 62.50 aphids/m^2^. Five species of primary parasitoids were recorded (*Aphidius sp., Diaretiella rapae, Aphidius ab senti, Trioxys sp*., and *Aphidius colemani*) from October 2009 to February 2010. The upper series of the bars in the diagram represent the primary parasitoids, and their relative abundance is indicated by the widths of each numbered bar. The mean density of primary parasitoids collected was 6.07 parasitoids/m^2^.

**Table 1.**
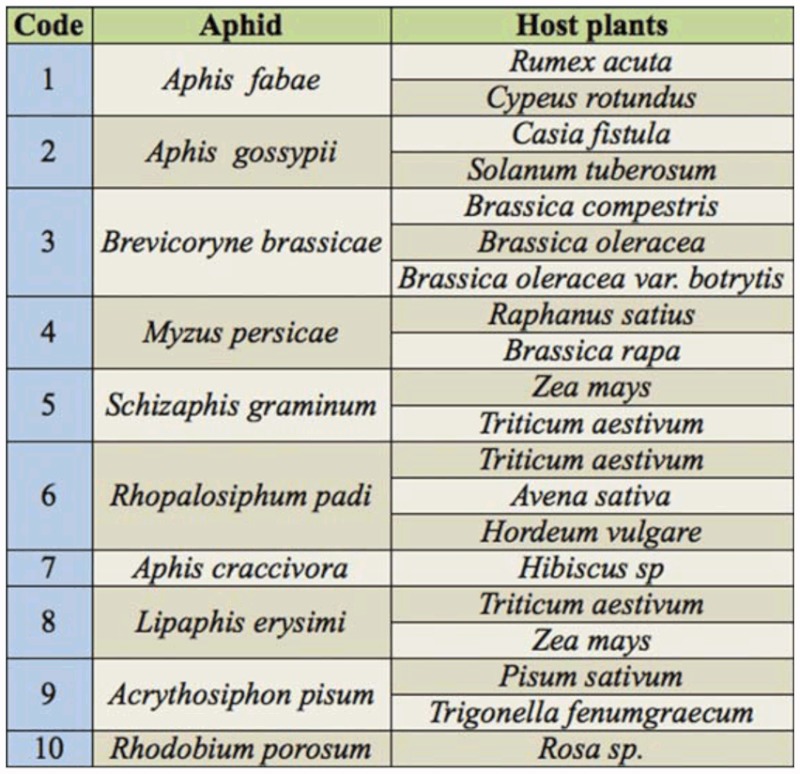
The field and horticultural crops upon which the aphids were collected.

**Table 2.**
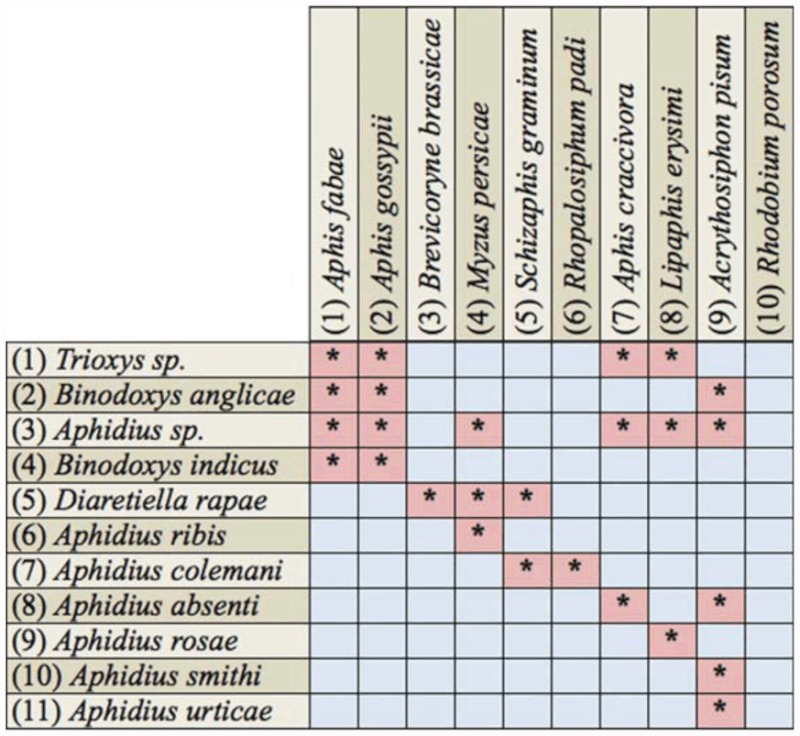
Host associations recorded during 2009–2010 (with codes for hosts and primary parasitoids in the parasitoid webs). Hosts (aphids) are listed across the top; the primary parasitoids are listed side of the table.

The parasitoid *Aphidius sp*. was reared from the aphid species *A. fabae, A. gossypii, M. persicae*, and *A. craccivora*; the parasitoid *D. rapae* was reared from *B. brassicae, M. persicae*, and *S. graminum*; the parasitoid *A. absenti* was reared from *A. craccivora*; the parasitoid *Trioxys sp.* was reared from *A. gossypii* and *A. craccivora*; and the parasitoid *A. colemani* was reared from *S. graminum* and *R. padi* in the Rawalpindi district (Figure 2).

The diversity of aphids in each district was calculated using the diversity index of Menhinick (1964). The maximum aphid diversity was found in Rawalpindi and the minimum diversity was in Jhelum (Table 3). A total of 5551 aphids and 761 mummified aphids were collected in Pothwar region (Table 4). The statistical analysis of different aphid species showed a significant difference between the population densities of aphids and the rate of parasitism as shown by mummified aphids (*F*_(9, 87)_ = 2.835, *p* < 0.01), (*F*_(9, 87)_ = 3.371, *p* < 0.01).

**Figure 7.**
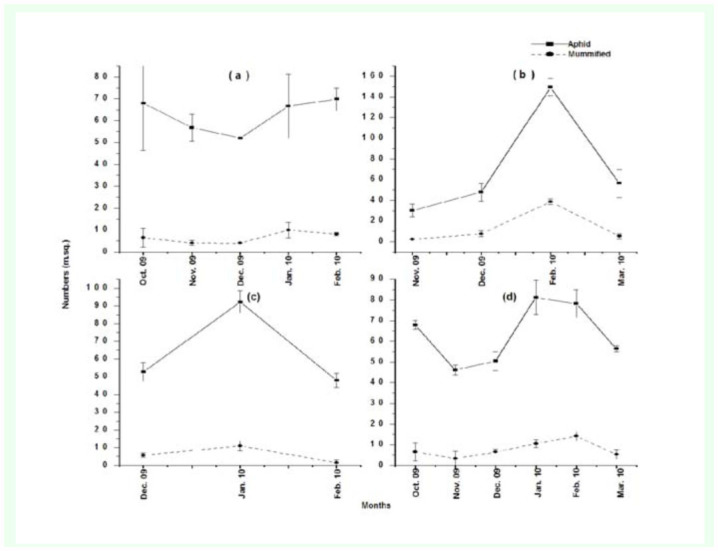
Population trend of aphids and mummified aphids in (a) Rawalpindi, (b) Chakwal, (c) Jhelum, and (d) Pothwar regions during 2009–2010. High quality figures are available online.

The maximum number of aphids was collected in January 2010 and the minimum number of aphids was collected in November 2009 (Figure 7). Similarly, the statistical analysis of monthly comparisons showed a significance difference between the population densities of aphids (*F*_(5, 87)_ = 3.830, *p* < 0.01), mummified aphids (*F*_(5, 87)_ = 4.736, *p* < 0.01), total plants (*F*_(5, 87)_ = 5.207, *P* < 0.01), and infested plants (*F*_(5, 87)_ = 7.908, *p* < 0.01).

**Table 3.**
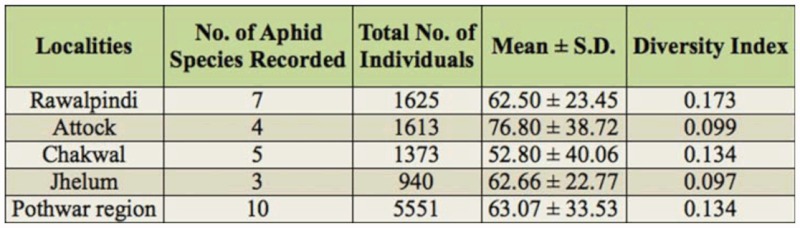
Diversity index of aphid species in Pothwar region during 2009–2010.

**Table 4.**
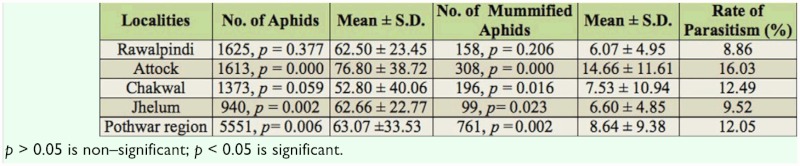
Number of aphids, mummified aphids, their means, and rate of parasitism found in Pothwar region during 2009–2010.

**Table 5.**
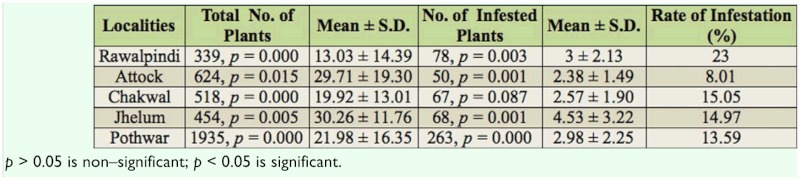
Rate of infestation found in Pothwar region during 2009–2010.

The maximum rate of parasitism was found in Attock, and the minimum in Rawalpindi (Table 4). These differences were correlated with Attock having the maximum mean number of mummified aphids (14.66 ± 11.61) per sampling unit, and Rawalpindi having the minimum mean number of mummified aphids collected (6.07 ± 4.95) per sampling unit. The overall rate of parasitism calculated in the Rawalpindi district was 12.05% (Table 4).

A total of 263 plants were infested with aphids, with a mean of 2.98 ± 2.25 infested plants per sampling unit and a rate of infestation of 13.59% in the Pothwar region (Table 5). The maximum infestation rate was in Rawalpindi and the minimum in Attock. The reason for these differences in infestation may be related to the rate of parasitism.

**Figure 8.**
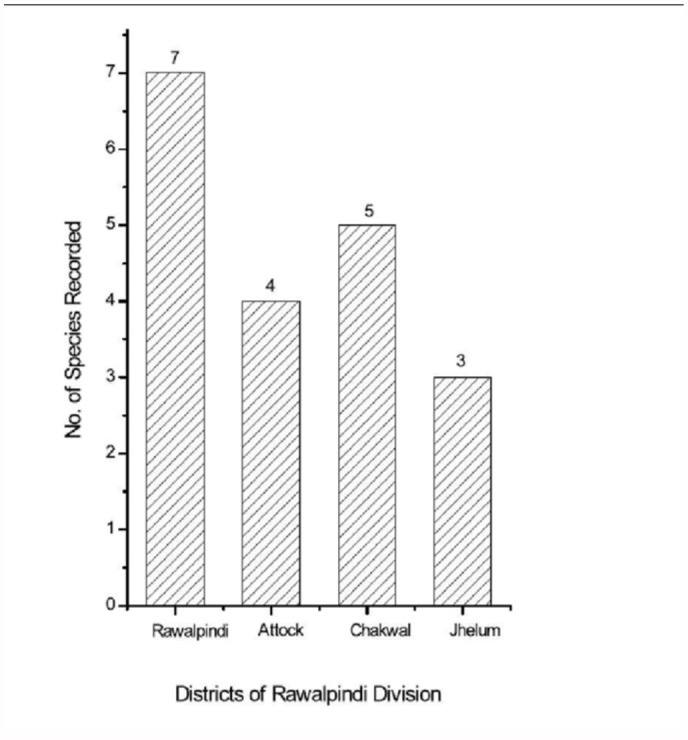
Species richness of aphids from various districts of Pothwar region. Maximum species of aphids collected from Rawalpindi and Minimum species collected from Jhelum. High quality figures are available online.

Species richness was highest in Rawalpindi with seven species present: *A. fabae, A. gossypii, B. brassicae, M. persicae, S. graminum, R. padi*, and *A. craccivora*. Only three species of aphids were collected from Jhelum (*B. brassicae, S. graminum*, and *R. padi*) and it had the lowest aphid species richness (Figure 8).

## Discussion

Five quantitative food webs were made describing the trophic relationship between the aphid and its primary parasitoid. In our study, the significant differences were found between the population densities of aphids, mummified aphids, and infested plants in Pothwar region in different months. The difference in the population trend of aphids may change many times throughout the season because of various factors, such as climatic conditions and natural enemy population. These two particular factors were considered to inhibit the aphid population from reaching outbreak stage (Irshad 2001; Bhambhro 2002). The population density of aphids was directly proportional to the population density of the mummified aphids. This was due to food availability for parasitoids and aphids (Abdullah 2006). In our study, the Rawalpindi district was rich in flora, and almost all the major field and horticultural crops were grown there, which might explain why the highest aphid diversity was recorded there. In contrast, Jhelum was less fertile, barren, and mountainous, which is unfavorable aphid species diversity as described by Zia et al. (2010). The maximum infestation rate was calculated in Rawalpindi and the minimum in Attock. The high infestation rate in Rawalpindi may be due to low parasitism rates, and the low infestation rate in Attock may be due to high parasitism rates. The host plants also influence both the population and performance of aphids (Dixon 1998). Many of the morphological defenses have been shown to have negative effects on aphids as they attempt to feed on the plants. Glandular hairs on plants can be an obstacle for aphids and other small arthropods. These factors can result in a low aphid population, and hence cause low infestation rates as described by Auclair (1989). The maximum species richness was found in Rawalpindi and the minimum in Jhelum. The distributions of aphid species vary because of geographical and host plants variations within the Rawalpindi district, resulting from ecosystems ranging from plainlands to mountains. Additionally, the use of different herbicides, especially on horticultural crops, induces changes in the plant community and affects arthropod abundance (Taylor et al. 2006).
